# CRISPR/Cas9 genome-wide screening identifies KEAP1 as a sorafenib, lenvatinib, and regorafenib sensitivity gene in hepatocellular carcinoma

**DOI:** 10.18632/oncotarget.27361

**Published:** 2019-12-17

**Authors:** Adi Zheng, Nadja Chevalier, Margot Calderoni, Gilles Dubuis, Olivier Dormond, Panos G. Ziros, Gerasimos P. Sykiotis, Christian Widmann

**Affiliations:** ^1^Department of Physiology, University of Lausanne, Lausanne, Switzerland; ^2^Service of Visceral Surgery, Lausanne University Hospital, Lausanne, Switzerland; ^3^Service of Endocrinology, Diabetology and Metabolism, Lausanne University Hospital, Lausanne, Switzerland; ^*^These authors contributed equally to this work

**Keywords:** KEAP1, sorafenib, hepatocellular carcinoma, lenvatinib, Regorafenib

## Abstract

Sorafenib is the first-line drug used for patients with advanced hepatocellular carcinoma (HCC). However, acquired sorafenib resistance in cancer patients limits its efficacy. Here, we performed the first genome-wide CRISPR/Cas9-based screening on sorafenib-treated HCC cells to identify essential genes for non-mutational mechanisms related to acquired sorafenib resistance and/or sensitivity in HCC cells. KEAP1 was identified as the top candidate gene by Model-based Analysis of Genome-wide CRISPR/Cas9 Knockout (MAGeCK). KEAP1 disrupted HCC cells were less sensitive than wild-type cells in short- and long-term sorafenib treatments. Compared to wild-type cells, KEAP1-disrupted cells showed lower basal and sorafenib-induced reactive oxygen species (ROS) levels and were more resistant to oxidative stress-induced cell death. The absence of KEAP1 led to increased activity of Nrf2, a key transcription factor controlling antioxidant responses, as further evidenced by increased expression of Nrf2-controlled genes including NQO1, GPX2 and TXNRD1, which were positively associated with chemoresistance. In addition, KEAP1 disruption counteracted the reduction of cell viability and the elevation of ROS caused by lenvatinib, a drug that recently showed clinical efficacy as a first-line treatment for unresectable HCC. Finally, Keap1 disruption also increased the resistance of cells to regorafenib, a recently approved drug to treat HCC as a second line therapy. Taken together, our data indicate that deregulation of the KEAP1/Nrf2 pathway following KEAP1 inactivation contributes to sorafenib, lenvatinib, and regorafenib resistance in human HCC cells through up-regulation of Nrf2 downstream genes and decreased ROS levels.

## INTRODUCTION

Hepatocellular carcinoma (HCC) is the most common type of primary liver cancer and the second most frequent cause of cancer death [1]. The only treatment available for advanced HCC is molecular targeted therapy using sorafenib (Nexavar^®^) or, since very recently, lenvatinib (Lenvima^®^). These drugs mainly act on serine-threonine kinases such as Raf-1 and on receptor tyrosine kinases such as vascular endothelial growth factor receptor (VEGFR) and platelet-derived growth factor receptor β (PDGFR-β) [2, 3], inhibiting angiogenesis, proliferation and tumor growth. Even though the median overall survival time is slightly higher in drug-treated patients compared to placebo [4, 5], the final prognosis remains dismal. The resistance that HCC acquires to these drugs is likely favored by genetic instability and heterogeneity leading to molecular and signaling alterations. For example, activation of the PI3K/Akt/mTOR pathway, inhibition of the JAK/STAT pathway, and increased autophagy have been associated with sorafenib resistance [6]. However, the mechanisms of sorafenib and lenvatinib resistance are still incompletely understood. Their elucidation may help to uncover molecular biomarkers to predict sensitivity to HCC treatment, as well as to identify new treatment strategies to overcome drug resistance. Recently, the CRISPR/Cas9 genome editing technology has been efficiently used to screen for genes involved in resistance to drugs. However, most studies are focused on parental cancer cells for initial drug response [7, 8]. Here, we established a sorafenib resistant cell line and performed a CRISPR/Cas9 screen to identify potential genes modulating acquired sorafenib resistance. This screen identified KEAP1 as a susceptibility gene to sorafenib.

## RESULTS

### A genome-wide CRISPR/Cas9 screening identifies KEAP1 as a gene involved in sorafenib sensitivity

**Figure 1:**
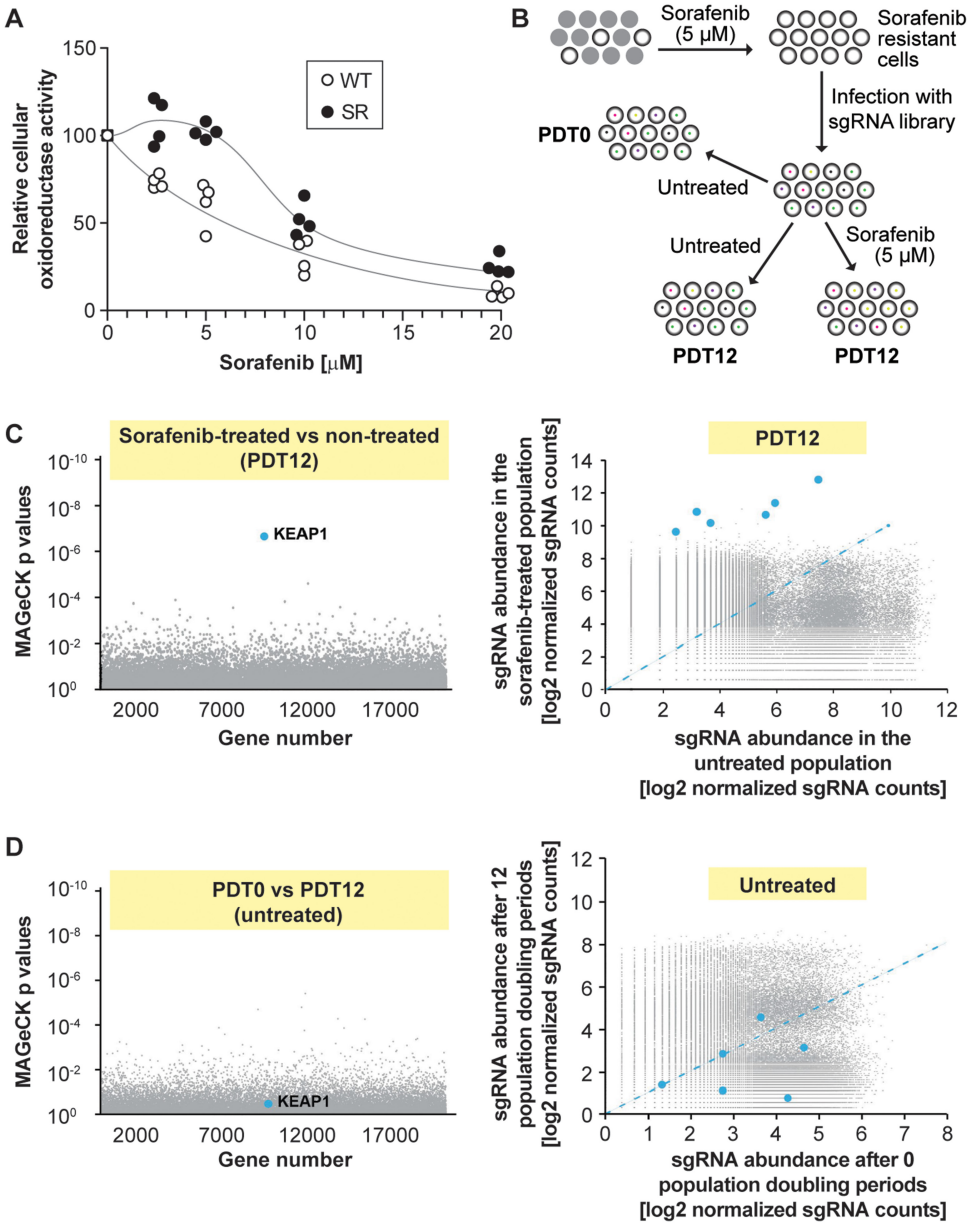
Single guide RNAs targeting KEAP1 are enriched in sorafenib-treated HUH-7 cells. (**A**) Sorafenib-resistant cells (SR) were derived from wild-type HUH-7 cells (WT) by three month exposure to 5 µM sorafenib. The WT and SR cells were treated with the indicated concentrations of sorafenib for 72 hours and viability was assessed by the MTS assay. Symbols are off set for clarity. The results were derived from four independent experiments. (**B**) Workflow of the screening process in HUH-7 cells. Cells were cultured in the presence of 5 µM sorafenib for 3 months. The resulting sorafenib-resistant population was infected with a sgRNA library (the vector used also encoded Cas9) and divided in three groups. The color dots in the cells indicate the presence of a given sgRNA. The first group was lysed immediately (population doubling time of 0; PDT0). The other two groups were allowed to proceed for 12 doubling times (PDT12) in the absence or in the presence of 5 µM sorafenib and then lysed. Twelve doubling periods have been deemed sufficient to induce depletion of sgRNAs that target genes required for a given resistance [41, 42]. After lysis, the abundance of the sgRNAs in the three groups was assessed by massive parallel sequencing (see Materials and Methods). (**C**) Identification of target genes involved in sorafenib resistance/susceptibility. The left panel depicts the *p* values associated with changes in sgRNA expression that were calculated using the MAGeCK procedure. The right panel shows a scatterplot of sgRNA abundance in sorafenib-treated vs control population after 12 doubling times. Blue dots correspond to the six KEAP1-targeting sgRNAs. The blue dashed line represents equal abundance of sgRNAs in both the untreated and treated populations. (**D**) As in (C) but comparing the untreated populations after 0 or 12 doubling times.

Our initial strategy was to use sorafenib-resistant (SR) HCC cell lines to identify genes modulating acquired resistance of the cells to the drug. For this purpose, SR HCC cell lines were derived from PLC/PRF/5, HepG2/C3A, and HUH-7 cells that were exposed during three months to 5 μM sorafenib, a clinically relevant dose [9]. Despite initial resistance to the drug obtained in the three cell lines, the resistance phenotype of PLC/PRF/5 and HepG2/C3A cells was not stable and was lost over time. In contrast, the resistance to sorafenib in HUH-7 cells was stable (Figure 1A); these resistant cells, called HUH-7 SR, were therefore used for the screening.

**Figure 2:**
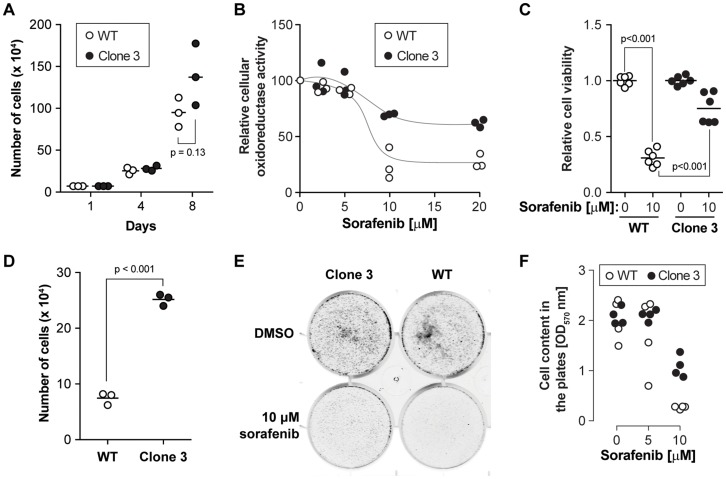
KEAP1 disruption decreases the sensitivity to sorafenib. (**A**) Cell proliferation of wild-type and KEAP1-disrupted cells. Cells were counted every three or four days. The results were derived from three independent experiments. (**B**) Cell viability was determined by MTS assay after exposure of the cells to different doses of sorafenib for 24 hours. The results were derived from three independent experiments. (**C**) Cell viability was determined by crystal violet assay on cells exposed or not to 10 µM sorafenib for 24 hours. The results were derived from four independent experiments (3 with one technical replicate and one with three technical replicates). (**D**) Cell number alteration in wild-type cells and disrupted cells after 5 µM sorafenib treatment in long-term response experiments (12 days). The results were derived from three independent experiments. (**E**) Clonogenic cell survival assay. Cells were treated with sorafenib at the indicated concentrations for two days. Then, 8,000 cells were seeded in new six well plates. Eight days later, cells were washed with PBS and stained with Crystal violet. (**F**) The quantitation of clonogenic growth was done by measuring the crystal violet content in the plates. The results were derived from four independent experiments.

**Figure 3:**
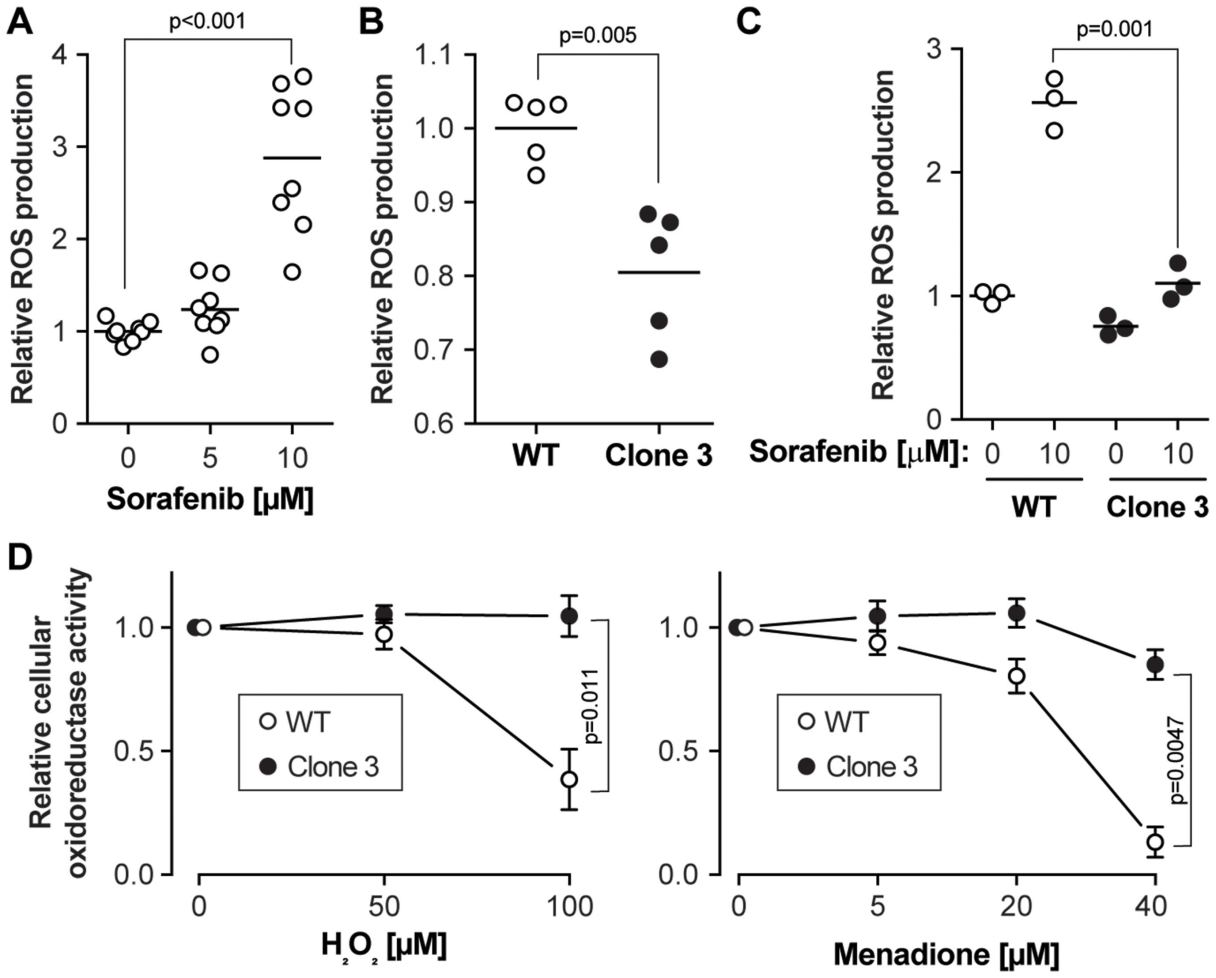
Sorafenib-induced ROS accumulation is reduced in KEAP1 disrupted cells. (**A**) Wild-type cells were exposed to different concentrations of sorafenib for 24 hours followed by ROS level measurement. The results were derived from four independent experiments (with two technical replicates each). (**B**) Basal ROS levels in wild-type cells and KEAP1 disrupted cells. The results were derived from two independent experiments (1 in duplicate and 1 in triplicate). (**C**) ROS levels in response to a 24 hour sorafenib stimulation in wild-type cells and KEAP1-disrupted cells. The results were derived from three independent experiments. (**D**) Cell viability after treatment with two different ROS inducers. The cell lines were treated with H_2_O_2_ or menadione for 7 hours at the indicated concentrations and cell viability was assessed using the MTS assay. Results correspond to the mean ± standard error of the mean. The results were derived from four independent experiments.

A CRISPR/Cas9-based genome-wide screening was used to identify genes in HUH-7 SR cells that conferred resistance to sorafenib [10], using the strategy shown in Figure 1B. Cells expressing the lentiviral sgRNA library were grown for 12 doubling times in the absence or in the presence of sorafenib. Cells expressing the library but not subjected to 12 doublings served as a control group. First, we looked for sgRNAs depleted in sorafenib-treated HUH-7 SR cells, because these could target genes required for the maintenance of sorafenib resistance. The rationale is that if a gene is required for sorafenib resistance, cells expressing the sgRNAs targeting this gene would have a survival or growth disadvantage in the presence of the drug. However, no significantly under-represented sgRNAs were identified and thus our screen failed to reveal an obvious gene candidate mediating the resistance phenotype in HUH-7 SR cells. In contrast, it was evident from the results that the six sgRNAs in the library targeting KEAP1 were significantly enriched in cells treated with sorafenib (Figure 1C). The 10 enriched genes with the highest enrichment of their corresponding sgRNAs following sorafenib treatment are listed in Supplementary Table 1. Among them, KEAP1 was the only gene with a FDR (false discovery rate) lower than 0.05. Increased expression of the KEAP1-targeting sgRNAs was not caused by a gene drift phenomenon that could occur when a cell population is cultured for long time periods, because there were no differences in the abundance of KEAP1-targeting sgRNAs between untreated cells analyzed before and after the 12 doubling time period (Figure 1D). As there seems to be a selective advantage to inactivate KEAP1 in the presence of sorafenib, the absence of KEAP1 is expected to confer increased proliferation or survival of HUH-7 cancer cells exposed to sorafenib. KEAP1 would correspond therefore to a sorafenib-sensitivity gene. The next set of experiments were designed to test this hypothesis. As KEAP1 does not contribute to the initial resistance of HUH-7 SR cells, these experiments were performed in the parental cells.

### KEAP1 invalidation decreases sorafenib sensitivity in HUH-7 cells

**Figure 4:**
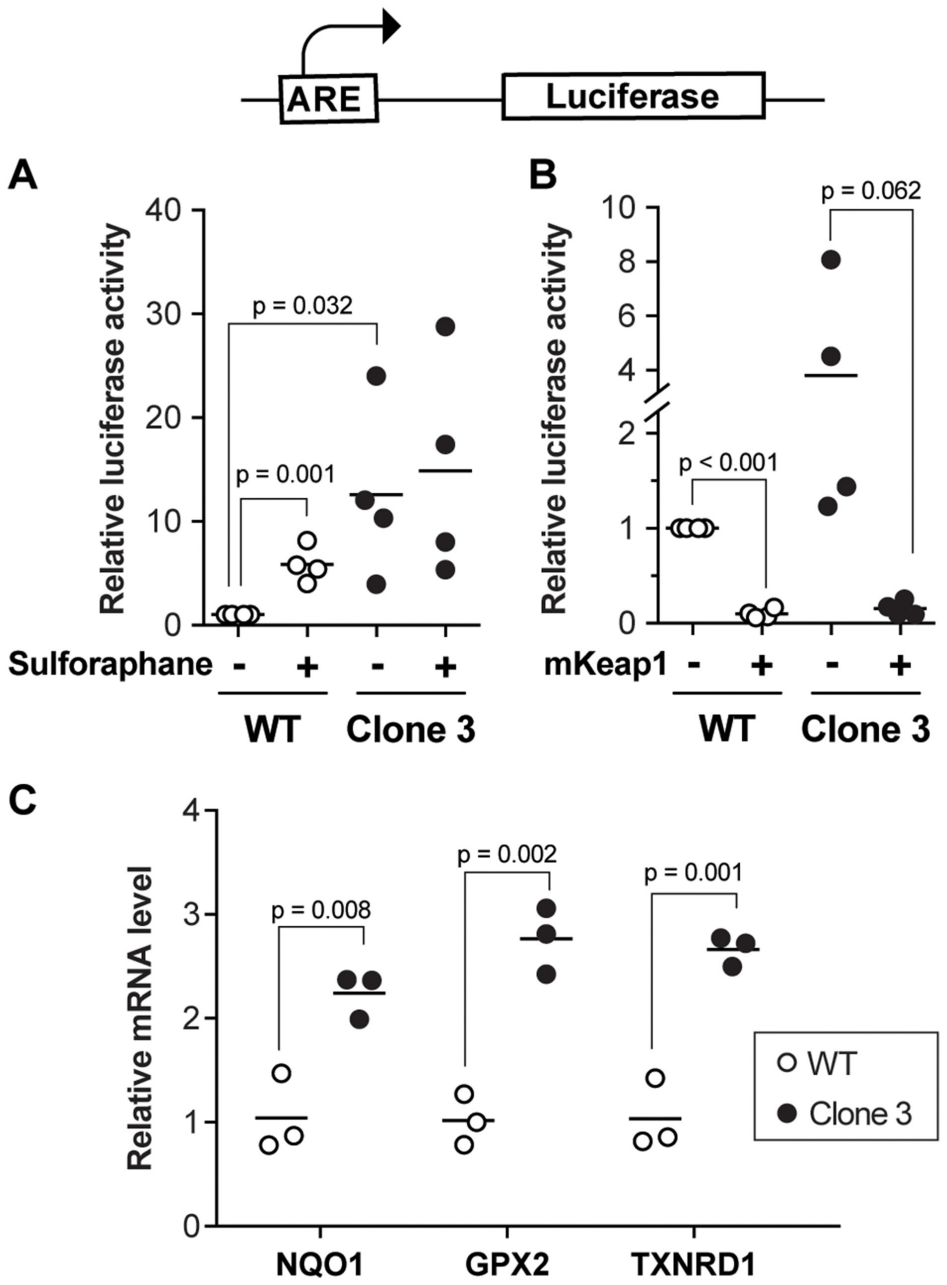
Defective regulation of Nrf2 target genes in KEAP1-disrupted cells. (**A**) ARE-driven luciferase activity was measured in wild-type and KEAP1-disrupted cells. Sulphoraphane (5 µM) was used as a positive Nrf2-inducing control. The results were derived from four independent experiments. (**B**) ARE-driven luciferase activity in wild-type cells and disrupted cells ectopically expressing or not mouse KEAP1. The results were derived from four independent experiments. (**C**) mRNA levels of Nrf2 target genes in wild-type and KEAP1-disrupted cells. The results were derived from three independent experiments.

To validate the role of KEAP1 in sorafenib susceptibility and to further investigate the role of KEAP1 in cells treated with the drug, we generated KEAP1 knockout HUH-7 cells. Using two different sgRNAs from the sgRNA library (Supplementary Figure 1A), we isolated two independent clones carrying different disrupting mutations in the KEAP1 alleles (Supplementary Figure 1B). The disrupted alleles in clone 3 encode slightly truncated versions of KEAP1. In clone 16, the disrupted alleles code for KEAP1 fragments that cannot be detected by the anti-KEAP1 antibody used here (Supplementary Figure 1A). KEAP1 functions as a repressor of Nrf2 activity [11]. Under basal conditions, KEAP1 binds to the ETGE and DLG motifs of Nrf2 and targets Nrf2 for Cul3-mediated ubiquitination, leading to Nrf2 proteasomal degradation [12]. In our screen, all six sgRNAs targeted the Kelch domain of KEAP1, which is responsible for the binding to Nrf2 [13]. Thus, the mutations introduced in the KEAP1 gene by these sgRNAs are all expected to affect its interaction with Nrf2, resulting in the stabilization of Nrf2 and the subsequent activation of its downstream target genes. This is indeed what was observed experimentally (Supplementary Figure 1D; see also Figure 4). Apparently the KEAP1/Nrf2 pathway was not affected in the SR cells compared to wild-type cells (Supplementary Figure 1C–1D). Despite similar ability to grow in control medium (Figure 2A and Supplementary Figure 3A), KEAP1-disrupted cells showed higher viability in the presence of high doses of sorafenib compared to control cells (Figure 2B–2C, Supplementary Figures 2 and 3B). In the presence of low doses of sorafenib (5 μM) for 12 days, cells lacking KEAP1 proliferated more than control cells (Figure 2D and Supplementary Figure 3C). The ability to form colonies in the presence of sorafenib was also higher in cells lacking KEAP1 (Figure 2E–2F). These results validate the screening data showing that KEAP1 is a sorafenib sensitivity gene in HUH-7 cells.

### Oxidative stress

Previous studies have shown that apart from targeting tyrosine and serine kinases, one possible anti-cancer mechanism of sorafenib in HCC is the induction of oxidative stress [14]. To investigate the oxidative stress levels in wild-type and KEAP1-disrupted cells, ROS levels were examined by H2DCFDA assay. This confirmed that sorafenib exposure led to ROS accumulation in wild-type HUH-7 cells (Figure 3A). Compared to wild-type cells, basal ROS levels were slightly decreased in KEAP1-disrupted cells (Figure 3B). In contrast to wild-type cells, sorafenib did not increase the ROS levels in the KEAP1 disrupted cells (Figure 3C). This indicates that the absence of KEAP1 activity in cells allows them to mitigate sorafenib-induced ROS accumulation. To further validate the role of the cellular antioxidant capacity in cell viability, wild-type and KEAP1-disrupted cells were treated with two different ROS inducers, H_2_O_2_ and menadione [15]. These inducers decreased cell viability in wild-type cells more than in KEAP1-disrupted cells (Figure 3D and Supplementary Figure 3D), confirming that KEAP1-disrupted cells are more resistant to drug-induced oxidative stress.

### The Nrf2 pathway is functional in HUH-7 cells

KEAP1 is a Cul3-based E3 ligase (Supplementary Figure 1A) that targets the Nrf2 transcription factor for degradation [16]. Nrf2 controls the expression of anti-oxidative genes, such as NQO1, through binding to antioxidant response elements (AREs) in their regulatory sequences, thereby increasing their basal and oxidative stress-inducible transcription [17, 18]. In our screen, all six sgRNAs targeted the Kelch domain of KEAP1, which is responsible for the binding to Nrf2. To investigate if KEAP1 disruption activates the Nrf2 pathway in our experimental setting, we first measured Nrf2 transcriptional activity by transfecting cells with a plasmid bearing the NQO1 promoter ARE sequence upstream of the luciferase reporter gene (Figure 4A). Compared to wild-type HUH-7 cells, KEAP1-disrupted cells showed markedly increased basal ARE-driven luciferase activity (Figure 4A). Sulforaphane is a well-characterized Nrf2-activating compound [19]. As expected, sulforaphane increased Nrf2 transcriptional activity in control HUH-7 cells but was unable to further augment the already high Nrf2 activity in KEAP1-disrupted cells (Figure 4A). The ability of the KEAP1-disrupted cells to repress basal Nrf2 activity was restored by forced over-expression of a sgRNA-resistant KEAP1 construct (Figure 4B), demonstrating that the defect in Nrf2 regulation observed in the KEAP1-disrupted cells was not a consequence of an off-target effect.

Nrf2 has a wide array of target genes, which are associated with several processes involved in cellular homeostasis maintenance. Therefore, the expression alterations of downstream target genes including NQO1, GPX2, and TXNRD1 between wild-type cells and KEAP1-disrupted cells were examined by real-time PCR. We found that NQO1, GPX2, and TXNRD1 were upregulated in KEAP1-disrupted cells as expected (Figure 4C and Supplementary Figure 3E). Previous studies have shown that high expression of Nrf2 target genes, including NQO1 [20], GPX2 [21], and TXNRD1 [22], is strongly associated with poor prognosis in HCC patients. These results further suggested that depletion of KEAP1 might contribute to HCC progression by increasing the transcription levels of Nrf2 target genes.

### The effects of KEAP1 disruption on lenvatinib and regorafenib sensitivity

**Figure 5:**
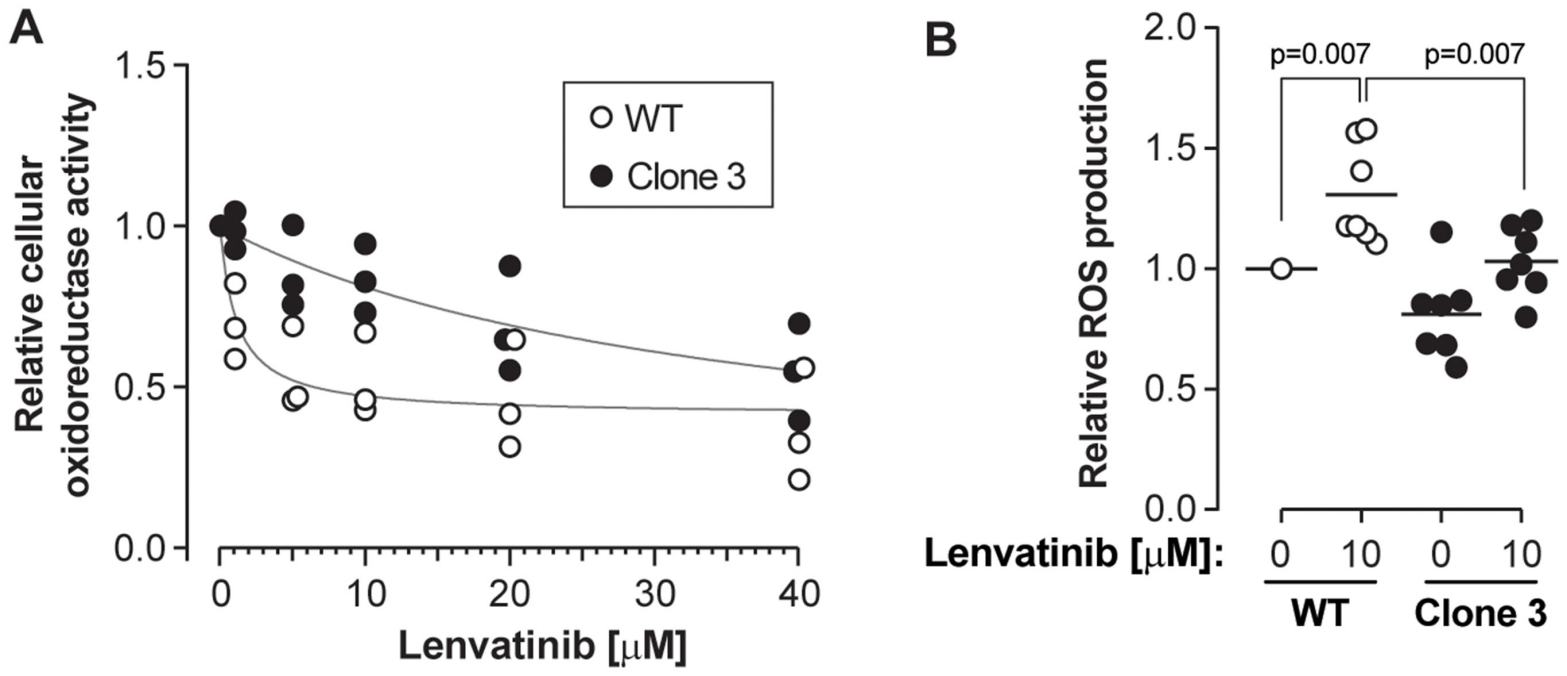
KEAP1 disruption confers resistance to lenvatinib. Cells were treated with the indicated concentrations of lenvatinib for 48 hours. (**A**) Cell viability was assessed using the MTS assay. The results were derived from three independent experiments. (**B**) ROS levels were measured as indicated in the Materials and Methods section. The results were derived from seven independent experiments.

Lastly, we tested whether KEAP1 disruption also decreased the sensitivity of HCC cells to lenvatinib, a drug that was shown recently to be as efficacious as sorafenib to treat HCC [5]. Like sorafenib, lenvatinib is a broad tyrosine kinase inhibitor, whose targets include VEGF receptors and FGF receptors [3]. To test the effects of lenvatinib, cells were treated with different concentrations of the drug for 48 hours. Figure 5A shows that KEAP1 disruption inhibited lenvatinib-induced drop in cell viability. This was accompanied by impaired ability to generate ROS in response to the drug (Figure 5B and Supplementary Figure 4). These data recapitulate those obtained for sorafenib, indicating that KEAP1 is also a lenvatinib susceptibility gene. Additionally, KEAP1-disrupted cells were also less sensitive to regorafenib (Stivarga^®^) (Supplementary Figure 5), a drug approved as a second-line therapy for patients progressing during or after sorafenib therapy [23].

## DISCUSSION

Previous studies have shown that sorafenib has a beneficial therapeutic effect on the treatment of HCC patients [4]. However, only a small fraction of HCC patients are sensitive to sorafenib and sorafenib resistance develops very often during treatment, limiting its utility [24]. Therefore, it is of importance to further elucidate the mechanisms related to sorafenib action and resistance. In our study, we demonstrated that the KEAP1 gene is involved in acquired sorafenib resistance using a genome-wide CRISPR/Cas9 screening. In HCC cells lacking KEAP1, basal and sorafenib-induced ROS levels were impaired. Furthermore, KEAP1 disruption led to increased Nrf2 activity and expression of Nrf2-driven genes. Our results indicate that KEAP1 disruption contributes to sorafenib resistance in human HCC cells through constitutive activation of the Nrf2 pathway.

Due to abnormal metabolism, cancer cells usually exhibit higher basal ROS levels compared to normal cells. They are therefore more sensitive to ROS-induced cell death [25]. This has led to the concept that ROS-induced tumor cell death is an important therapeutic strategy in cancer treatment. Here, we confirmed that ROS levels were increased by sorafenib in wild-type HUH-7 cells, but not in KEAP1-disrupted cells. At the same time, KEAP1-disrupted cells were more resistant to oxidative stress-induced proliferation impairment and death. This suggests that loss of KEAP1 causes sorafenib resistance through suppression of cellular oxidative stress.

The KEAP1/Nrf2 pathway is a major regulator of cellular responses to oxidative stress. Western blot and luciferase assay demonstrated that Nrf2 transcriptional activity was induced in KEAP1 disrupted HUH-7 cells. A number of ARE-containing genes are regulated by Nrf2, which can be divided into 5 groups: antioxidant enzymes, NADPH-generating enzymes, metal-binding proteins, drug-metabolizing enzymes and drug transporters, and stress response proteins [26]. Some of them have been reported to impact drug resistance in different kinds of cancers [27, 28]. In HCC patients, aberrant activation of this pathway can result from Nrf2 mutations in or adjacent to the DLG and ETGE motifs or by KEAP1 inactivation [29–31]. High expression levels of several Nrf2-targeted genes are strongly correlated with poor survival in HCC patients [20, 22]. Our results showed that the mRNA levels of NQO1, GPX2 and TXNRD1 were upregulated in KEAP1-disrupted cells. Collectively, these data suggest that Nrf2 activation mediates KEAP1 loss-of-function-induced resistance to sorafenib and lenvatinib through regulation of ROS level in HCC.

One limitation of this study is that only one hepatocarcinoma cell line (HUH-7 cells) was investigated. Further work on additional HCC cell lines might therefore be required to extent the findings of the present paper. In this context however, it is worth mentioning that a recent study has indeed demonstrated that sorafenib resistance can be conferred through decreased KEAP1 protein expression leading to increased Nrf2 activity in HCC cell lines distinct from HUH-7 cells [32]. Using an unbiased genome-scale CRISPR/Cas9-based screening in sorafenib resistant cells, we now independently identify KEAP1 as a sorafenib-sensitivity gene in both resistant and wild-type HCC cells and further extended these findings to the recently approved anti-HCC drugs, lenvatinib and regorafenib.

An interesting effect was sometimes observed (see Figure 1A for example): in response to very low doses of sorafenib or lenvatinib HUH-7 cells tended to display increased viability (or growth or mitochondrial metabolism) compared to non-treated cells. This can be related to earlier work showing that low non-lethal stresses can paradoxically favor cell survival. One proposed mechanism for such responses is that low stresses induce mild caspase-3/7 activity, leading to partial cleavage of the p120 RasGAP protein into a potent anti-apoptotic N-terminal fragment [33].

The findings on KEAP1 that we are reporting here have important clinical implications. Firstly, as KEAP1 loss mediates sorafenib, lenvatinib, and ragorafenib resistance, we suggest that patients with HCC that are resistant to first-line treatment with sorafenib, if mediated by KEAP1 loss-of-function, will not respond to second-line treatment with lenvatinib or ragorafenib (and vice-versa). KEAP1/Nrf2 pathway inactivation (through KEAP1 or Nrf2 mutations for example) can be a poor prognostic factor for HCC treatment and help clinicians predict the characteristics of specific cancers. Clinical translational studies are warranted to test these hypotheses, which are critical to guide patient management and especially to avoid treatment failures, systemic side-effects, delays and costs from ineffective drug choices.

Secondly, the requirement of KEAP1/Nrf2 pathway deregulation for sorafenib resistance in both parental and acquired resistant HCC cells provides a potential intervention target for future therapies. Aberrant Nrf2 activation occurs not only in HCC cancer but also in many other types of cancer [34, 35]. Sustained Nrf2 activation promotes the survival and proliferation of cancer cells in hepatocarcinogenesis [36]. Therefore, Nrf2 inhibitors, which are currently under development [37], may be effective in synergistic therapy to combat HCC and prevent or overcome drug resistance. Moreover, the importance of the KEAP1/Nrf2 pathway in both acquired and primary sorafenib resistance, suggests that Nrf2 inhibitor could be beneficial not only in the initial treatment stages targeting primary tumors, but also in later stages to eliminate acquired resistant cells. These concepts could be tested as soon as Nrf2 inhibitors become available.

## MATERIALS AND METHODS

### Cell culture

The human liver carcinoma HUH-7 cell line and its derivatives were maintained at 37°C in a humidified incubator with 5% CO_2_ in DMEM medium (Gibco, Paisley, UK) supplemented with 10% fetal bovine serum (FBS) (Invitrogen, ref. no. 10270-106), 100 U/ml penicillin, and 100 μg/ml streptomycin (Gibco, Paisley, UK).

### Reagents

Sorafenib was obtained from LC laboratories (ref. no. S8502). Lenvatinib was purchased from Selleck Chemicals (ref. no. S1164). 2’7’-dichlorodihydrofluorescein diacetate (H2DCFDA), H_2_O_2_ solution and menadione were purchased from Sigma (ref. no. D6883, H1009, and M2518). Lipofectamine 2000 reagent and sulforaphane were obtained from Invitrogen (ref. no. 11668019) and Enzo Life Science (ref. no. ALX-350-230-M010), respectively. SDS (20%) was obtained from Fisher bioreagents (ref. no. bp1311-1). Nrf2 antibody and KEAP1 antibody were purchased from Abcam (ab62352) and Cell Signaling (#8047S), respectively.

### MTS/PMS cell metabolic activity assay

This assay records the NAD(P)H-dependent cellular oxidoreductase enzyme activity, a proxy for cell viability. Cells were plated in 96-well plates and incubated overnight. Cells were then treated as indicated in the figures. At the end of the treatments, the cells were incubated with a MTS/PMS (3-(4,5-dimethylthiazol-2-yl)-5-(3-carboxymethoxyphenyl)-2-(4-sulfophenyl)-2H-tetrazolium inner salt)/phenazine methosulfate) solution (Promega ref. no. G5421) for 1 hour at 37°C. The optical density was measured using the Cytation 3 cell imaging multi-mode reader (BioTek Instruments) at a wavelength of 490 nm.

### Genome-scale CRISPR/Cas9-mediated knockout screen

The human GeCKO v2 library (2-plasmid system) (Addgene ref. no. 1000000049) was amplified in Endura bacteria (Lucigen, ref. no. 60242) by electroporation using a Gene Pulser II electroporation system (Bio-Rad, ref. no. 165-2105). Cells were plated on LB Agar plates containing 100 μg/ml ampicillin. After 14 hours at 32°C, colonies were scrapped and plasmids recovered with the Plasmid Maxi kit from Qiagen (ref. no. 12162). To produce the lentivirus library, 12 T-225 flasks were seeded with 12 × 10^6^ HEK293T cells/flask. The following day, each flask was transfected, using the calcium/phosphate transfection method, with 10 μg pMD2.G (#554), 30 μg psPAX2 (#842) and 25 μg of the GeCKO plasmid library. The plasmids were diluted in a 250 mM CaCl_2_ solution. This solution was then mixed (v/v) with 2x HEPES buffer (NaCl 280 mM, KCl 10 mM, Na_2_HPO_4_ 1.5 mM, D-glucose 12 mM, HEPES 50 mM), incubated for 1 minute at room temperature and then added to the culture medium. Seven hours later, the medium was removed and replaced by DMEM supplemented with 10% FBS, 100 U/ml penicillin, and 100 μg/ml streptomycin. Forty-eight hours later, the medium was collected and centrifuged for 5 min at 2,000 g to pellet the cells. The supernatant was filtered through a 0.45 μm HV/PVDF (Millipore, ref. no. SE1M003M00) and concentrated 100x by ultracentrifugation at 70,000 g for 2 hours at 4°C. Virus pellet was resuspended in ice-cold PBS, aliquoted and stored at –80°C.

The Cas9 endonuclease was stably expressed in HUH-7 SR cells following infection with a lentivirus produced using the lentiCas9-Blast plasmid (Addgene, ref. no. 52962) (#849) and a one-week selection in the presence of 35 μg/ml blasticidin. The multiplicity of infection (MOI) of the GeCKO virus library was determined as follows: different volumes of the virus library were added to 250,000 Cas9-expressing HUH-7 SR cells plated in a 6-well plate. Twenty-four hours later, each well was split into duplicate wells and one well received 10 μg/ml puromycin for 3 days. Cell viability was determined by trypan blue exclusion. The minimal volume of viruses leading to ~100% survival was considered as the conditions giving a MOI of 1. For the CRISPR/Cas9 screen a MOI of 0.3 was chosen (to insure that the majority of cells are not infected with more than one virion). Large scale infection of 12 × 10^7^ HUH-7 SR cells was carried out in 6-well plates with 250,000 cells per well. Twenty-four hours later, wells were pooled in a T-225 flask and infected cells selected with 10 μg/ml puromycin for a week. After puromycin selection, the cells were split into two parts. One part (6 × 10^7^ cells) was left untreated. The other part (6 × 10^7^ cells) was treated with 5 μM sorafenib for 12 population doublings with sorafenib-containing medium renewals every 3 days. After 12 doubling times, control and sorafenib-treated surviving cells were collected, and their genomic DNA was extracted with the Blood & Cell Culture DNA Midi Kit (Qiagen, ref. no. 13343). Twenty million cells were also collected, and their genomic DNA extracted, just after the puromycin selection (hence not submitted to the 12 doublings).

A first PCR was performed to amplify the lentiCRISPR sgRNA region with primers F1 and R1 (Table 1). A second PCR was performed to attach Illumina adaptors and barcodes to samples. Five μl of the first PCR product were used. Primers for the second PCR included both a variable length sequence to increase library complexity and a 6 bp barcode for multiplexing of different biological samples (F2a-R2_iA_12, Table 1). Both PCRs were performed in 100 μl with the Herculase II Fusion DNA Polymerase (Agilent, ref. no. 600675). Amplicons were gel extracted, quantified, mixed and sequenced on a MiSeq instrument (Illumina). Raw FASTQ files were demultiplexed and processed to contain only unique sgRNA sequences. The number of reads of each sgRNA was normalized as described [7]. The MAGeCK algorithm [38] was used to rank screening hits by consistent enrichment among multiple sgRNAs targeting the same gene.

**Table 1:** Primers used in the CRISPR/Cas9-based screening

**F1**	AATGGACTATCATATGCTTACCGTAACTTGAAAGTATTTCG
**R1**	CTTTAGTTTGTATGTCTGTTGCTATTATGTCTACTATTCTTTCC
**F2a**	AATGATACGGCGACCACCGAGATCTACACTCTTTCCCTACACGACGCTCTTCCGATCT*TCTTGTGGAAAGGACGAAACACCG*
**F2b**	AATGATACGGCGACCACCGAGATCTACACTCTTTCCCTACACGACGCTCTTCCGATCT***AGC*** *TCTTGTGGAAAGGACGAAACACCG*
**F2c**	AATGATACGGCGACCACCGAGATCTACACTCTTTCCCTACACGACGCTCTTCCGATCT***CGAGC*** *TCTTGTGGAAAGGACGAAACACCG*
**F2d**	AATGATACGGCGACCACCGAGATCTACACTCTTTCCCTACACGACGCTCTTCCGATCT***CATAACC*** *TCTTGTGGAAAGGACGAAACACCG*
**F2e**	AATGATACGGCGACCACCGAGATCTACACTCTTTCCCTACACGACGCTCTTCCGATCT***GTGCTAACG*** *TCTTGTGGAAAGGACGAAACACCG*
**R2_iC_26**	CAAGCAGAAGACGGCATACGAGAT**GCTCAT**GTGACTGGAGTTCAGACGTGTGCTCTTCCGATCT*TCTACTATTCTTTCCCCTGCACTGT*
**R2_iA_12**	CAAGCAGAAGACGGCATACGAGAT**TACAAG**GTGACTGGAGTTCAGACGTGTGCTCTTCCGATCT*TCTACTATTCTTTCCCCTGCACTGT*

Bold: barcodes; Italic bold: variable length sequence; Italic: sequence annealing to the amplicons from the first PCR.

### KEAP1 gene disruption

Gene disruption of the KEAP1 gene was performed using the CRISPR/Cas9 technology as described [39]. The sequences of the sgRNA used for this are shown in Supplementary Figure 1B.

### Cell proliferation

Seventy thousand wild-type HUH-7 cells and KEAP1-disrupted HUH-7 cells were separately seeded in 60 mm dish and treated with 5 µM sorafenib for 12 days. Medium was changed every three or four days. Cell number was counted using a hemocytometer.

### Measurement of cell death using propidium iodide (PI)

Eighty thousand wild-type HUH-7 cells and KEAP1-disrupted HUH-7 cells were separately seeded in six well plates and treated as indicated in the figures. All cells including floating cells in the medium were collected and suspended in 500 µl PBS solution containing PI (8 µg/ml). Cell death was measured by flow cytometry using a Beckman Coulter FC-500 apparatus. PI was excited with 20 mW of an argon ion laser (488 nm). For forward scatter measurement, the voltage was set at 425 mV with a gain of 1 Amp. For side scatter measurement, the voltage was set at 129 mV with a gain of 5 Amp.

### Reactive oxygen species (ROS) measurement

H2DCFDA was used to detect ROS generation. Cells were cultured in six-well plates. Following the treatments indicated in the figures, cells were washed once with PBS and 10 μM H2DCFDA was added to the cells at 37°C in the dark. Thirty minutes later, cells were washed with PBS and lysed in 10 mM Tris, 150 mM NaCl, 0.1 mM EDTA and 0.5% Triton X-100, pH 7.5. The supernatant (40 µl) was assayed using a Cytation 3 cell imaging multi-mode reader with an excitation wavelength of 495 nm and an emission wavelength of 538 nm. The concentration of total protein was determined by the Bradford technique. ROS levels were normalized against the protein content (fluorescence [arbitrary units] divided by microgram of protein).

### Crystal violet assay

Cells in 6-well plates or 35 mm Petri dishes were washed once with PBS, air-dried for 10 minutes, and fixed with absolute ethanol for 10 more minutes. Cells then were stained with 0.05% crystal violet (in water/0.5% ethanol) for 30 minutes. The plates were gently washed with water twice and air-dried for 10 minutes. For quantitation of this staining, the cell-associated crystal violet was dissolved in 1.5 ml of 1% SDS in water. Two aliquots of 200 µl per sample were placed in a 96-well plate and then the optical density was measured with the Cytation 3 cell imaging multi-mode reader (BioTek Instruments) at a wavelength of 570 nm. The optical density recordings for a given sample were averaged.

### Antioxidant response element (ARE) luciferase reporter assay

Cells (100,000 in 500 μl) were seeded in 24-well plates. The following day, cells were transfected with a luciferase reporter plasmid (#998) driven by an ARE sequence from the NAD(P)H quinone dehydrogenase 1 (NQO1) promoter [40] using Lipofectamine 2000 for 48 hours. The pEGFP plasmid (#6) coding for green fluorescent protein was co-transfected to assess transfection efficiency. After treatment with or without 5 µM sulphoraphane for 24 hours, GFP fluorescence was measured using a Cytation 3 cell imaging multi-mode reader with an excitation wavelength of 480 nm and an emission wavelength of 520 nm. To measure the luciferase reporter activity, a luciferase reporter assay (Promega, UK) was performed according to the manufacturer’s protocol. Luciferase activities were normalized to GFP fluorescence. Relative luciferase activity is expressed as fold change over wild-type control.

### Real-time PCR

Total RNA was extracted from cells using TRIzol reagent (Invitrogen). Reverse transcription was performed using the Transcriptor Universal cDNA Master kit from Roche, followed by semiquantitative real-time PCR with the indicated primers listed in Table 2. Data were analyzed by the 2^-ΔΔCt^ method and normalized to the GAPDH PCR amplicon. Relative expression of genes is expressed as fold change over control.

**Table 2:** Primers used for real-time PCR

Gene	Forward	Reverse
GAPDH	CTGACTTCAACAGCGACACC	TGCTGTAGCCAAATTCGTTG
NQO1	ATGTATGACAAAGGACCCTTCC	TCCCTTGCAGAGAGTACATGG
GPX2	CTGGTGGTCCTTGGCTTC	GTTCTGCCCATTCACCTCAC
TXNRD1	CCACTGGTGAAAGACCACGTT	AGGAGAAAAGATCATCACTGCAGAT

### Statistics and data presentation

Comparisons between multiple groups were performed using one-way ANOVA followed by Sidak’s multiple comparisons test using GraphPad Prism. Comparisons between two groups were performed using Student’s *t*-test or Sidak’s multiple comparisons test in GraphPad Prism. Horizontal bars in the figure correspond to the mean. When normalization is shown in figures, the individual replicates of a given experiment (including those of the control group) are normalized against the average value of the control group for this given experiment.

## SUPPLEMENTARY MATERIALS



## Abbreviations

KEAP1: Kelch-like ECH-associated protein 1
Nrf2: Nuclear factor erythroid 2-related factor 2
GPX2: Glutathione peroxidase 2
TXNRD1: Thioredoxin reductase 1
