# Supplementary material for: CRISPR/Cas9 genome-wide screening identifies KEAP1 as a sorafenib, lenvatinib, and regorafenib sensitivity gene in hepatocellular carcinoma
Source: Oncotarget. 2019 Dec 17;10(66):7058–70. doi: 10.18632/oncotarget.27361 (PMC6925031; doi:10.18632/oncotarget.27361)
Supplement: Supplementary file 1 [file oncotarget-10-7058-s001.pdf]

CRISPR/Cas9 genome-wide screening identifies KEAP1 as a sorafenib, lenvatinib, and regorafenib sensitivity gene in hepatocellular carcinoma

SUPPLEMENTARY MATERIALS

Supplementary Table 1: The top 10 genes the sgRNAs of which displayed the highest enrichment following sorafenib selection

ID	Number of sgRNAs in the library	*P*-value	FDR
KEAP1	6	2.42E-07	0.00495
NIPA1	6	2.54E-05	0.259901
DMTF1	6	0.000133	0.757426
MAD2L2	6	0.000156	0.757426
C1orf50	6	0.000185	0.757426
PPAP2C	4	0.000266	0.826733
hsa-mir-381	4	0.000283	0.826733
DYNC1LI1	6	0.00033	0.844678
CASP8AP2	6	0.000485	0.869931
RBFOX2	6	0.000526	0.869931

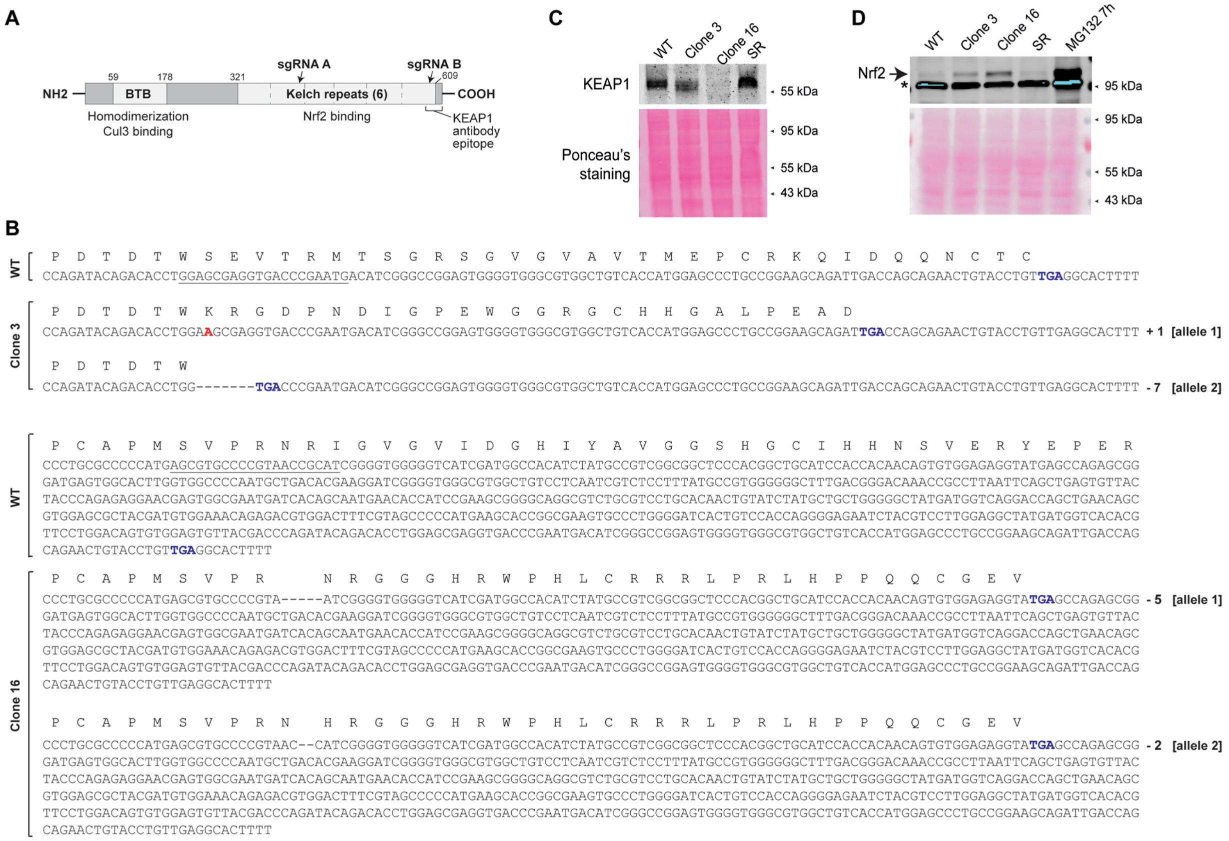

Supplementary Figure 1: KEAP1 disruption. (**A**) Schematic representation of the KEAP1 protein and some of its domains. The BTB domain participates in KEAP1 homodimerization and Cul3 binding, while the Kelch repeats are involved in Nrf2 binding. The epitope recognized by the KEAP1 antibody used in this study is located at the extreme C-terminus of the protein. BTB, Broad complex, Tramtrack and Bric-à-Brac. Clone 3 and clone 16 were obtained from disruption of the Keap1 gene alleles using sgRNA B and sgRNA A, respectively. (**B**) DNA sequences of wild-type and KEAP1 mutant alleles. The location of the KEAP1 sequences targeted by the sgRNAs used to generate the KEAP1-invalidated clones are underlined. The stop codons and the insertions are marked in bold (blue for the stop codons and red for the insertions). Deletions are indicated by dashes. (**C–D**) The expressions of KEAP1 (C) and Nrf2 (D) in control wild-type cells (WT), KEAP1-disrupted cells and sorafenib-resistant cells (SR) were evaluated by western blot analysis. In panel D (last lane), WT cells were treated with 10 μM of the MG132 proteasome inhibitor for 7 hours as a means to increase Nrf2 expression so that its expression could be better visualized. The Nrf2 protein band is marked by an arrow. *, non-specific band.

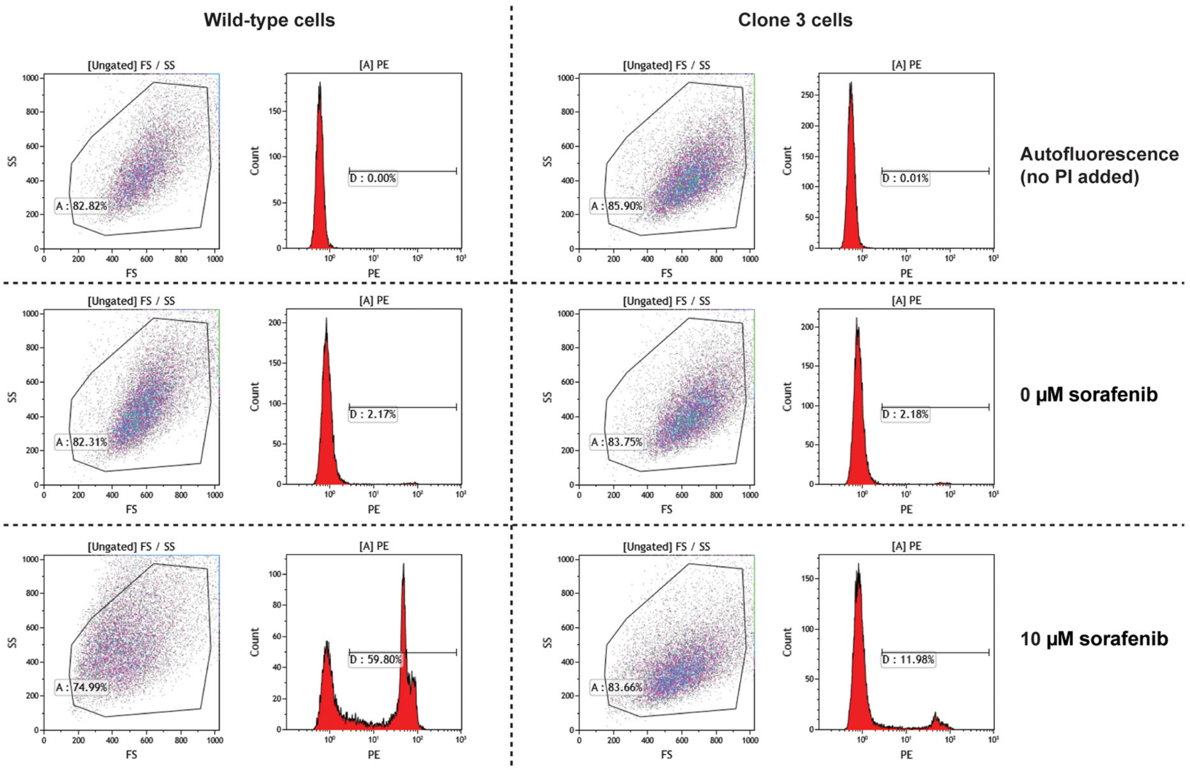

Supplementary Figure 2: Cell death induced by sorafenib in short-term experiment. Wild-type cells and KEAP1-disrupted cells were incubated with the indicated sorafenib concentration for 24 hours. Cell death was then measured by PI staining. Autofluorescence was determined in untreated cells not incubated with PI.

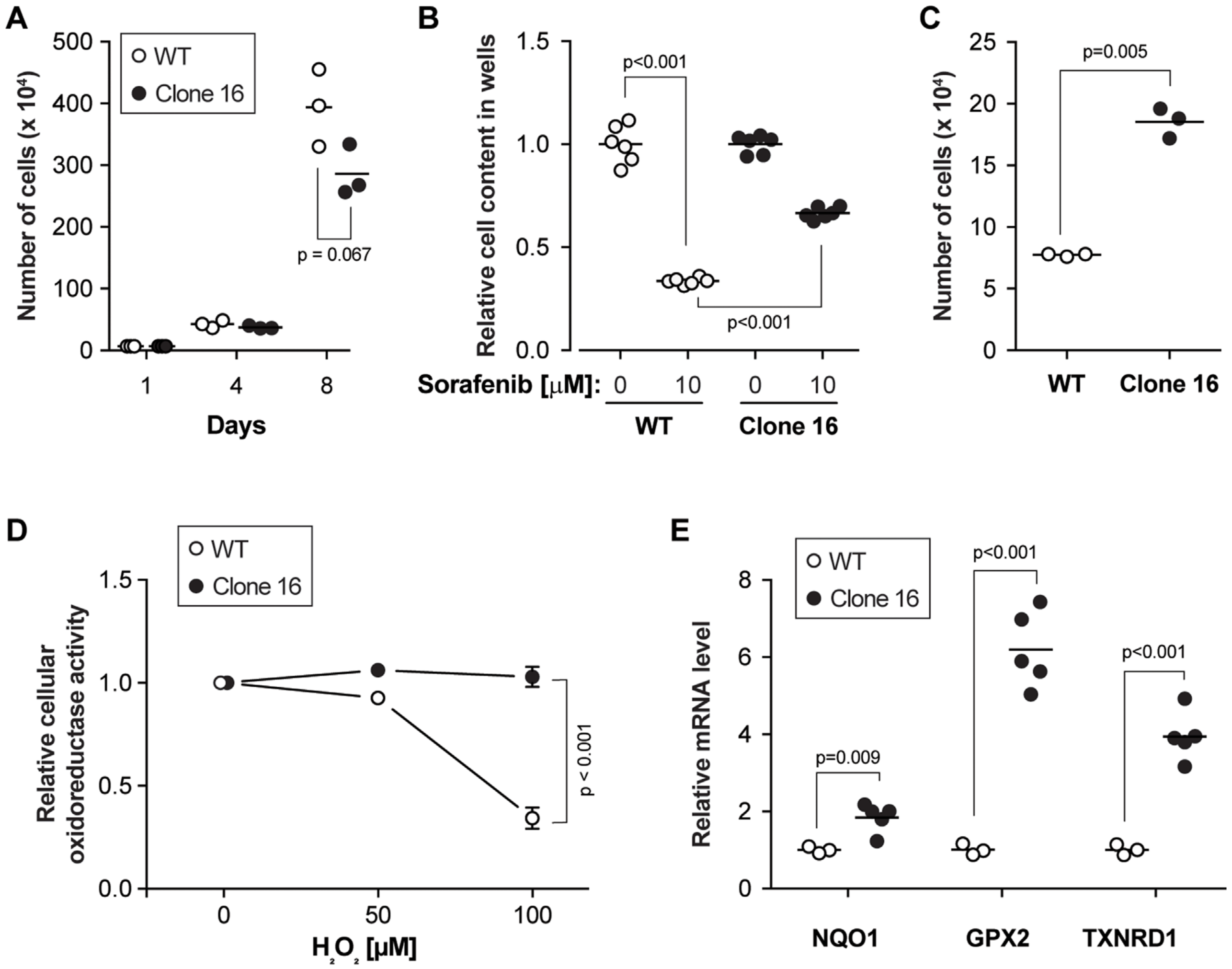

Supplementary Figure 3: Sorafenib resistance in clone 16. (**A**) Cell proliferation in wild-type cells and KEAP1-disrupted cells (clone 16). Cells were counted every three or four days. The results were derived from three independent experiments. (**B**) Cell content in wells was determined by crystal violet assay on cells exposed or not to 10 µM sorafenib for 24 hours. The results were derived from four independent experiments (3 performed in triplicate and 1 with one technical replicate). (**C**) Cell numbers of wild-type and KEAP1-disrupted cells after 5 µM sorafenib treatment in long-term response experiments (10 days). The results were derived from three independent experiments. (**D**) Cell viability after treatment with ROS inducers. Cells were treated with H_2O_2 for 7 hours at the indicated concentrations and cell viability was assessed by the MTS assay. Results correspond to the mean ± standard error of the mean. The results were derived from four independent experiments (2 technical replicates each). (**E**) mRNA levels of Nrf2 target genes in wild-type and clone 16. The results were derived from three independent experiments.

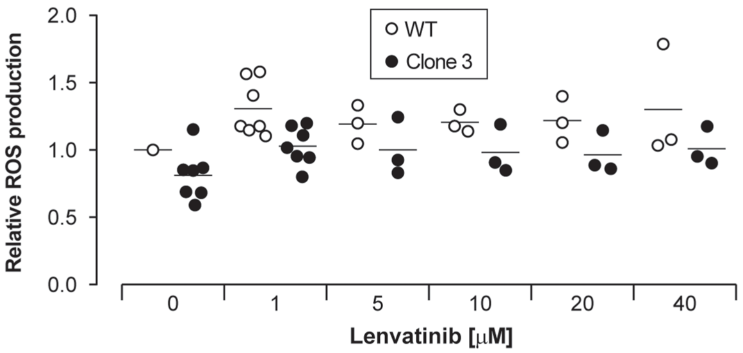

Supplementary Figure 4: ROS levels after lenvatinib treatment. Wild-type cells and KEAP1-disrupted cells (clone 3) were treated with increasing concentrations of lenvatinib for 48 hours. ROS levels were measured as indicated in the Methods. The results were derived from three independent experiments.

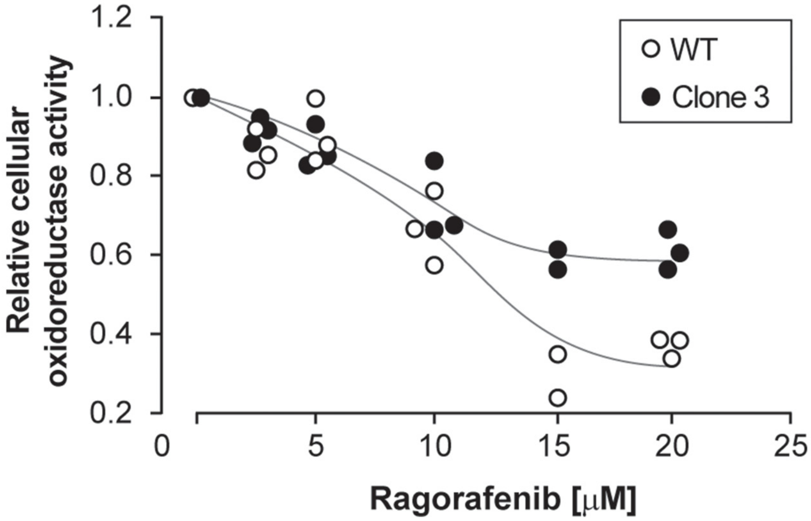

Supplementary Figure 5: KEAP1 disruption decreases sensitivity to regorafenib. Wild-type cells and KEAP1-disrupted cells (clone 3) were treated with increasing doses of regorafenib for 24 hours. Cell viability was assessed using the MTS assay. The results were derived from three independent experiments.
